# 铂类抗肿瘤药物相关肾损伤作用机制的研究进展

**DOI:** 10.3779/j.issn.1009-3419.2015.09.09

**Published:** 2015-09-20

**Authors:** 建春 段

**Affiliations:** 100142 北京，北京大学肿瘤医院（北京大学临床肿瘤学院）胸部肿瘤内一科 Department of Thoracic Medical Oncology, Beijing Cancer Hospital and Institute, Peking University, Beijing 100142, China

**Keywords:** 铂类药物, 肾损伤, 作用机制, Platinum derivatives, Kidney injury, Mechanism

## Abstract

铂类药物是目前应用最广泛的抗肿瘤药物之一，广泛应用于卵巢癌、睾丸肿瘤、头颈部肿瘤、肺癌和结直肠癌等恶性实体瘤中，然而，其严重的不良反应以及耐药问题限制了其临床应用。同时该类药物可引起较严重的不良反应，从而限制了铂类药物的临床应用范围。其中，限制顺铂使用的最主要因素是肾脏毒性。本文就不同铂类药物引起肾损伤的作用机制进行综述。

## Abstract (en)

Platinum derivatives are the most widely used chemotherapeutic agents to treat solid tumors including ovarian, head and neck, and testicular germ cell tumors, lung cancer, and colorectal cancer. Two major problems exist, however, in the clinic use of platinum derivatives. One is the development of tumor resistance to the drug during therapy, leading to treatment failure. The other is the drug's toxicity such as the cisplatin's nephrotoxicity, which limits the dose that can be administered. This paper describes the mechanism of platinum derivatives induced kidney injury.

铂类药物是迄今为止应用最广泛的抗肿瘤药物之一，其通过跨膜转运进入细胞，解离失去酸根负离子，作用于DNA分子，导致DNA结构改变，DNA复制转录障碍，引起细胞凋亡。自1978年第一个铂类药物——顺铂（cisplatin, DDP）上市以来，其就被广泛应用于多种恶性实体瘤的治疗中，具有抗肿瘤谱广、有效性高的特点，但使用仍受到各种限制，限制因素包括原发或继发的肿瘤耐药性，以及严重的不良反应，如：肾毒性、消化道反应、耳毒性、神经毒性等，其中最主要剂量限制性毒性是肾毒性。为了寻找更安全高效的化疗药物，在过去的30多年里，人们从23种铂类抗肿瘤药物中挑选出了2种（卡铂和奥沙利铂）并通过国际市场批准，另外亦有3种铂类抗肿瘤药物（奈达铂、洛铂和庚铂）在少数国家上市。迄今为止，还有4种铂类抗肿瘤药物（赛特铂、吡铂、Lipoplatin^TM^和ProLindac^TM^）处于不同的临床研究阶段^[1]^。

因大部分药物及其代谢产物最终经肾脏排出，所以药物引起的肾损伤发生较为常见。药物引起的肾损伤发生率高达18.3%，其中抗生素肾损害的发生率达36%^[2]^。然而，也有资料^[3]^显示，近年来抗生素所导致的肾损伤逐渐减少，而非甾体抗炎药、血管紧张素转换酶抑制剂、化疗药及抗病毒药物所致的肾损伤却在增加。在铂类药物中，DDP是第一代铂类药物，其肾毒性是剂量累积和剂量限制性毒性，尽管在临床上已经采取水化利尿、剂量调整、明确用药禁忌证等措施来预防肾损伤的发生，但仍有4%-23%的患者会出现急性肾损伤（acute kidney injury, AKI）^[4]^。卡铂（carboplatin, CBP）和奈达铂（nedaplatin, NDP）是第二代铂类药物，虽然两者肾毒性明显降低，但约10%的患者在使用CBP过程中出现血肌酐升高，尤其是当CBP每天使用剂量 > 1, 750 mg时，发生肾损伤的风险明显升高^[5]^。在关于NDP的一项Ⅰ期临床研究中，只观察到少数患者血尿素氮和肌酐一过性轻度升高，没有观察到明显的肾损伤^[6]^。奥沙利铂（oxaliplatin, OXA）是第三代铂类药物，耐受性良好，关于OXA引起肾毒性的报道较少见，在一项Ⅰ期临床研究中，血肌酐2级（世界卫生组织化疗药物毒副反应分级标准）升高的发生率为4%^[7]^。虽然广大医务工作者一直致力于研究能够减轻肾损伤的药物或措施，比如充分的水化、γ-氨基丁酸^[8]^、补充镁剂^[9]^等，但这些药物治疗效果欠佳，只能部分减少肾损伤的发生。本文就DDP、CBP、NDP、OXA引起肾损伤的不同机制进行总结，以指导临床用药，提高抗肿瘤疗效，同时尽可能减少肾毒性发生。

## 1 顺铂

DDP，又名顺-双氯双氨络铂，于1969年开始用于临床。DDP在血浆中呈电中性，基本不发生解离，大部分以与血浆蛋白结合的形式存在。DDP进入细胞后，由于氯离子浓度下降，DDP发生解离并与DNA直接结合形成DNA链内或链间交联，干扰DNA的复制，引起细胞不可逆损伤，乃至死亡，属周期非特异性药物。有基础研究^[10]^发现DDP在肾脏特异性的累积是引起肾毒性的重要原因。

### 1.1 DDP的细胞摄取

DDP主要通过3个途径进入细胞：被动扩散、转运蛋白介导的易化扩散和主动吸收^[11]^，其中主动吸收在顺铂耐药及毒性的发生中起非常重要的作用。参与铂类易化扩散和主动吸收的膜转运蛋白包括：铜离子转运蛋白（copper transporters, Ctrs）、有机阳离子转运蛋白（organic cation transporters, ocTs）、溶质载体（solute carriers, SLCs）及三磷酸腺苷结合的多种药物转运蛋白（adenosine triphosphate-binding cassette, ABC）等^[12]^。当铜离子转运蛋白1（copper transporter 1, Ctr1）缺失时，酵母菌对DDP的摄取减少^[13]^，与DDP准运相关的近端肾小管细胞凋亡也减少^[14]^。在Ctr1表达增多时，DDP、CBP、OXA细胞内累积量增加^[15, 16]^。在DDP的摄取中，ocTs也起到非常重要的作用^[17]^。ocTs为肾脏有机阳离子转运体家族之一，包括有机阳离子/肉毒碱转运体（organic cation/carnitine transporters, ocTNs）、多药及毒素外排转运体（multidrug and toxin extrusion transporters, MATEs）和多药耐药蛋白1（multidrug resistance proteins 1, MDR1）等^[18]^。ocTs由可溶性载体22基因家族编码表达，ocT1、ocT2氨基酸序列的相同度达70%，在人体中，ocT1主要在肝脏表达，ocT2主要在肾脏内表达，ocT3分布较广，但在肾中微量表达。MATEs包括3个亚家族（rMate1/hMATE1、hMATE2、rMATE2），MATE1和MATE2氨基酸序列的相同度为47.5%，后者又被克隆出2种：MATE2-K和MATE2-B，但目前MATE2-B未发现功能；MATE2-K与MATE2氨基酸存在94%的相似度^[19]^。ocT2抑制剂——西咪替丁可以减少体外培养的肾小管细胞对DDP的摄取，抑制DDP引起的细胞凋亡^[20]^。在过表达ocT2的人近端肾小管细胞中DDP摄取量和毒性均明显增加^[20]^。此外，在缺乏ocT1/ocT2的小鼠中则未出现DDP引起的肾小管损伤^[21, 22]^。当ocT2表达增加时，OXA的细胞摄取量和毒性增加。当ocT3表达增加时，OXA的细胞摄取量和毒性反而减少，而在CBP和NDP中却没有发现该现象^[23]^。最近研究^[24]^发现，在对DDP敏感的宫颈癌KB-3-1细胞系中ocT3表达增多，提示ocT3也可能参与DDP的转运。Yonezawa等^[23]^还发现MATE1可以增加HEK293细胞系中DDP的累积量，且比MATE2-K增加的幅度大，而对于OXA细胞累积量来说，MATE2-K比MATE1增加的幅度大。由此可见，铂类抗肿瘤药物进入细胞是多种转运蛋白参与的，在不同的组织细胞中各种转运蛋白所起到的作用也不尽相同。

### 1.2 肾脏损伤的病理生理特征

DDP引起AKI的病理生理特征是：①近端肾小管损伤；②氧化应激；③炎症反应；④肾脏血管损伤。近端肾小管的损伤包括几种不同的作用机制，包括细胞凋亡、细胞自噬、细胞周期蛋白调节异常、丝裂原活化蛋白激酶信号通路激活、对肾小管上皮细胞直接损伤、DNA损伤、线粒体功能异常^[25]^等。

#### 1.2.1 近端肾小管损伤

DDP的剂量直接影响细胞的死亡类型^[26]^。在体外实验中，高浓度的DDP导致细胞坏死，而低浓度的DDP诱导细胞凋亡，而在动物试验中，DDP既可以引起细胞坏死又可以诱导细胞凋亡^[27]^。细胞凋亡途径包括通过死亡受体介导的外源性通路和以线粒体、内质网应激通路为主的内源性通路。在外源性通路中，DDP激活Fas（APO-1/CD95）和肿瘤坏死因子-α（tumor necrosis factor α, TNF-α）等死亡受体，进一步激活下游的半胱氨酸蛋白酶（caspases），最终引起细胞凋亡^[28]^。在内源性通路中，细胞损伤引起Bcl-2蛋白相关X蛋白（Bcl-2 associated X protein, Bax）和Bcl-2蛋白拮抗剂（Bcl-2 antagonist/killer, Bak）激活，引起包括细胞色素C、凋亡诱导因子（apoptosis-inducing factor, AIF）、第二线粒体来源的半胱氨酸天冬氨酸蛋白水解酶激活剂或低等电点的凋亡蛋白抑制剂家族直接结合蛋白（second mitochondrial activator of caspases/direct inhibitor of apoptosis protein-binding protein of low isoelectric point, Smac/DIABLO）和线粒体内核酸内切酶G等因子释放，进一步激活半胱天冬蛋白酶-9（caspase-9），最终导致细胞凋亡^[29]^。抑制Bax后可以减少线粒体损伤和DDP引起的凋亡的发生^[30]^。值得注意的是，在动物实验中发现，当*Bax*基因缺失时，老鼠表现出对DDP耐药^[31]^。在内质网应激凋亡通路中，DDP可以激活caspases通路的起始因子——caspase-12。细胞转染抗caspase-12抗体后，细胞凋亡受抑^[32]^。抑制内质网磷脂酶A2也可减少DDP引起的近端肾小管上皮细胞凋亡^[33]^。此外，DDP引起AKI时会伴有p53磷酸化，当抑制p53后，肾小管上皮细胞凋亡、肾脏组织损伤以及DDP引起的AKI发生减少^[34]^。

肾小管上皮细胞暴露于DDP中后，细胞自噬的标志物包括自噬基因Beclin1，微管相关蛋白1轻链3（microtubule-associated protein 1 light chain 3，MAPlLC3，简称LC3），自噬相关基因5（autophagy-related gene 5, Atg5）明显升高^[34]^。虽然目前细胞自噬对于整个细胞周期来讲尚有争议^[35]^，但在DDP引起的AKI中，细胞自噬起到保护作用。人们发现，肾小管上皮细胞暴露于DDP后，细胞自噬可以促进细胞存活，延缓细胞凋亡的启动^[36]^。在抑制细胞自噬后，DDP引起的AKI加重。雷帕霉素（rapamycin, RAPA）可通过抑制哺乳动物雷帕霉素靶蛋白（mammalian target of rapamycin, mTOR）通路，诱导和促进细胞自噬的发生，可以减少DDP所致AKI的发生^[37]^。此外，DDP还可以激活肾小管上皮细胞炎症信号通路分子p38丝裂原活化蛋白激酶（p38 mitogen-activated protein kinase, p38-MAPK）细胞外信号调节激酶（extracellular signal-regulated kinase, ERK），c-Jun氨基末端激酶/应激激活的蛋白激酶（c-Jun N-terminal kinases/stress activated protein kinases, JNK/SAPK）等通路，抑制细胞外信号调节激酶1/2（extracellular signal-regulated kinases 1/2, ERK1/2）通路，调节细胞周期，影响线粒体功能和细胞凋亡等^[25]^。

#### 1.2.2 氧化应激

肾脏中活性氧族（reactive oxygen species, ROS）的产生、脂质过氧化物的累积和抗氧化系统被抑制是DDP引起AKI的主要作用机制之一。DDP进入肾小管细胞后可与含巯基的抗氧化剂谷胱甘肽反应^[38]^，降低其细胞内浓度，使细胞内氧化应激反应增加。DDP也可以引起线粒体功能障碍，使呼吸链受损，并促进ROS的产生^[39]^。ROS增加导致脂质过氧化，使细胞膜结构和通透性改变，影响细胞功能。ROS还可损伤氨基酸、蛋白质和碳水化合物，促进DNA损伤和细胞凋亡^[40]^。

#### 1.2.3 炎症反应

研究^[41]^发现，在DDP引起的AKI中，许多炎症因子包括IL-1β、IL-6、IL-18、单核细胞趋化蛋白-1（monocyte chemotactic protein-1, MCP-1）、调节活化正常T细胞表达与分泌的趋化因子（regulated upon activation normal T cell expressed and secreted, RANTES）、巨噬细胞炎症蛋白-2（macrophage inflammatory protein-2, MIP-2）、细胞间粘附分子-1（intercellular cell adhesion molecule-1, ICAM-1）、转化生长因子-β（transforming growth factor beta, TGF-β）表达增多。但在小鼠中抑制IL-1、IL-6及IL-18后，AKI的发生并未减少。

Caspase-1是一种促进炎症反应的细胞因子，caspase-1可以使IL-1β、IL-18活化。在敲除caspase-1基因的（caspase-1-/-）小鼠中，应用DDP后导致AKI减少。但是单纯抑制IL-1β和IL-18并不能减少AKI的发生^[41]^，目前机制未明。TNF-α在DDP导致的AKI过程中也起到了非常重要的作用。DDP可以使血浆和尿液中TNF-α增加。抑制TNF-α可以使其他的炎症因子和趋化因子包括IL-1β、MCP-1、RANTES减少^[42]^。经TNF-α抑制剂处理或敲除*TNF-α*基因的小鼠，接受DDP注射后AKI发生减少^[42]^。TNF-α抑制剂水杨酸盐类药物可以减少普通小鼠中接受DDP注射时引起的AKI，但是在敲除*TNF-α*基因的小鼠中却没有此保护作用^[43]^。Kim等^[44]^也发现，TNF-α抑制剂己酮可可碱也可减少DDP相关肾毒性的发生。此外，IL-33也是介导DDP引起AKI过程的促进炎症反应的炎症因子，而IL-10却可以减少DDP相关肾毒性的发生^[25]^。

#### 1.2.4 肾脏血管损伤

血管内皮功能紊乱和血管自动调节受损引起的肾血管收缩是DDP引起AKI的另一重要机制。DDP可以引起急性缺血损伤，伴肾髓质血流量减少，进而引起肾小管细胞受损^[45]^。DDP可以直接损伤血管内皮细胞，低浓度时引起细胞凋亡，高浓度时导致细胞坏死^[46]^。此外，血浆中血管假性血友病因子（von Willebrand factor, vWF）在血管内皮受损时会增多^[47]^，研究^[48]^发现在DDP引起肾损伤出现之前血浆中vWF升高且达峰值，这也说明DDP会引起血管内皮的损伤。

### 1.3 顺铂肾损伤的防治

顺铂主要通过直接损伤肾小管、肾血管、氧化应激以及炎症反应等机制引起AKI，目前临床上主要通过水化利尿来减少AKI的发生，并通过检测肌酐、尿素氮等指标来监测肾功能。此外，针对顺铂引起肾损伤的不同作用机制的药物也应运而生，比如：氮氧化物4-羟基-2, 2, 6, 6-四甲基哌啶（tempol）是一种超氧化物歧化酶类似物，通过抑制氧化应激，减少线粒体损伤在不影响化疗疗效的同时减少AKI的发生^[49]^。沙利度胺可以通过抑制炎症反应而减少肾损伤的发生^[50]^。钩吻素甲具有抗炎抗肿瘤活性的作用，通过阻断脂质过氧化，抑制黄嘌呤氧化酶活性，增加过氧化氢酶、谷胱甘肽过氧化酶、谷胱甘肽还原酶等抗氧化物质，减少顺铂引起的肾损伤的发生^[51]^。此外，在一项回顾分析研究^[52]^中发现，一般状态较差、规律使用非甾体类抗炎药物的患者更容易出现顺铂引起的肾损伤，而使用镁剂的患者肾损伤发生率则降低。然而上述研究大部分仅限于体外细胞或体内动物模型中，仍缺乏临床应用的相关数据，其疗效仍是未知。

## 2 卡铂

CBP全称是顺式-1, 1-环丁烷二羧酸二氨铂。与DDP相比，具有如下特点：①化学性质稳定，水溶性是DDP的17倍；②其主要剂量限制性毒性是骨髓抑制、肾毒性、胃肠道反应明显低于DDP，患者耐受性好；③作用机制与DDP相同，可以代替DDP用于某些癌症的治疗，与DDP有交叉耐药；④与非铂类药物无交叉耐药性，可以联合用药^[53]^。虽然与DDP相比，CBP肾毒性明显减低，并且可以应用于终末期肾病需要行血液透析治疗的患者^[54]^，但仍有10%的患者会在治疗过程中出现血肌酐升高，尤其是当存在以下高危因素时：如联合使用其他肾毒性药物、高龄、吸烟、女性、低白蛋白血症等，其肾毒性增加。此外，核苷酸切除修复交叉互补基因1（excision repair cross-complementing 1, *ERCC1*）和*TP53*的遗传基因多态性也会影响肾毒性的发生^[55]^。

CBP主要通过肾小球滤过和肾小管分泌排出体外，其代谢产物可在肾脏累积，引起肾小管损伤^[56]^。CBP引起肾损伤的机制尚未完全清楚，但推测可能与肾脏中自由基、活性氧增加及抗氧化因子减少有关^[57]^。人们通过研究CBP对小鼠肾脏的影响发现，卡铂引起的肾脏病理生理改变与范可尼综合征相似，可出现肾性糖尿、多种氨基酸尿、高钙尿症，且己酮可可碱——非特异的磷酸二酯酶抑制剂可以减少肾损伤的发生^[58]^。此外，普伐他汀也可减少CBP导致肾损伤的发生^[59]^。

## 3 奈达铂

NDP又名为顺式-乙醇酸-二氨合铂，于1995年在日本首次获准上市，目前国内已获批上市，但尚未广泛使用。与DDP相比，其水溶性约为DDP的10倍，其主要剂量限制性毒性是骨髓抑制，肾毒性明显低于DDP，与DDP有交叉耐药。在关于NDP的一项Ⅰ期临床研究中，只观察到少数患者血尿素氮和肌酐呈一过性轻度升高，未观察到明显的肾毒性^[6]^。NDP主要引起肾乳头的损伤，包括肾乳头坏死，集合管上皮细胞和肾乳头被覆上皮细胞透明样变，细胞凋亡和再生性增生、肾小管扩张以及轻微的肾皮质内近端肾小管和远端肾小管的损伤^[60]^。这可能与NDP与近端肾小管，包括ocT2和多药及有毒化合物排出家族（multidrug and toxic compound extrusion, MATE）在内的转运蛋白亲和力较低有关^[61]^。此外，当给予小鼠同样剂量的NDP和DDP时，前者在肾脏的累积量仅为DDP的40%^[62]^。给予水化可以减少NDP引起肾毒性的发生^[63]^。

## 4 奥沙利铂

OXA又名左旋反式二氨环己烷草酸铂，于1996年首次在法国上市。其疗效好、毒性低，而且与DDP、CBP无交叉耐药。主要剂量限制性毒性是外周神经病变，关于OXA相关肾损伤的报道比较少见。在一项关于OXA的Ⅰ期临床研究中，血肌酐一过性升高达世界卫生组织（World Health Organization, WHO）不良反应分级2级的发生率仅有4%^[7]^。近几年关于OXA肾损伤的个案报道逐渐增多。Joybari等^[64]^通过总结相关的个案报道，发现OXA可引起急性肾小管坏死（3例）、肾小管酸中毒（3例）、溶血性尿毒综合征（1例）、范可尼综合征（2例）、肾小管空泡形成（1例）及免疫介导性溶血性贫血引起的AKI（6例）。OXA引起肾损伤的机制尚未研究清楚。最近研究^[23]^发现，当ocT2表达增加时，OXA的细胞摄取量和毒性增加，MATE也可以使OXA细胞内累积量增加，且MATE2-K比MATE1所致细胞内累积量增加的幅度更大。但亦有相反的研究结论：Yokoo等^[65]^发现，MATE可以介导OXA从肾小管细胞流出，起到肾脏保护的作用。此外个体差异亦可能影响OXA对肾脏的损伤。

## 5 总结

铂类药物是细胞周期非特异性抗肿瘤药物，广泛应用于多种恶性实体肿瘤的治疗中，具有有效率高、抗瘤谱广的特点，但严重的不良反应限制了铂类药物的使用，例如DDP的肾毒性、CBP和NDP的骨髓抑制以及OXA的周围神经损伤。与DDP相比，其余3种铂类药物的肾毒性明显减低，这主要与顺铂在肾脏的特异性累积有关。此外，不同铂类药物引起肾脏损伤的病理改变也不尽相同：DDP主要累及近端肾小管，CBP可引起类似范可尼综合征的病理改变，NDP主要引起肾乳头的损伤，关于OXA的肾毒性目前还处于个案报道阶段。此外，在不同实体瘤中，不同铂类药物的有效率有所差异，所以，在选择铂类药物时，既要考虑药物的毒副反应，又要结合化疗有效率进行综合分析，制定个体化治疗方案。目前，人们一方面致力于研究高效低毒的新型铂类抗肿瘤药物，另一方面也试图通过调整剂量、选择合适患者、采取水化利尿和具有肾保护作用的药物等措施来减少肾毒性的发生，但效果仍有限。所以，只有在了解不同铂类药物引起不同的肾损伤类型，尤其是特殊类型的肾损伤后，才能更好地在不影响肿瘤治疗疗效的同时早期识别、及时处理甚至减少肾损伤的发生。
